# Safety of immune checkpoint inhibitors in cancer patients with COVID-19: A review

**DOI:** 10.1097/MD.0000000000043579

**Published:** 2025-08-08

**Authors:** Kejian Yang, Haixiang Xie, Zuyin Wan, Xin Zhou, Junqi Liu, Jusen Nong, Jianzhu Luo, Chongjiu Qin, Tao Peng

**Affiliations:** a Department of Hepatobiliary Surgery, The First Affiliated Hospital of Guangxi Medical University, Nanning, Guangxi Zhuang Autonomous Region, People’s Republic of China; b Guangxi Key Laboratory of Enhanced Recovery After Surgery for Gastrointestinal Cancer, Nanning, Guangxi Zhuang Autonomous Region, People’s Republic of China; c Key Laboratory of Early Prevention & Treatment for Regional High Frequency Tumor (Guangxi Medical University), Ministry of Education, Nanning, Guangxi Zhuang Autonomous Region, People’s Republic of China.

**Keywords:** cancer patients, COVID-19, immune checkpoint inhibitors, immune-related adverse events

## Abstract

Coronavirus disease 2019 has emerged as a substantial burden to global public health, with cancer patients exhibiting heightened susceptibility to severe complications. Immune checkpoint inhibitors have exhibited noteworthy efficacy in cancer therapy by promoting robust anti-tumor immune responses. Nevertheless, the safety and efficacy during epidemics remain contentious. The extant evidence concerning the persistent administration of immune checkpoint inhibitors in cancer treatment within the context of the coronavirus disease 2019 epidemic has been consolidated in this review, and the significance of rigorous patient screening and vigilant monitoring has been emphasized to equilibrate anticancer efficacy with the risk of immune dysfunction, thereby establishing a foundation for the research in cancer immunotherapy.

## 1. Introduction

Since the onset of the coronavirus disease 2019 (COVID-19) outbreak, a rapid global spread has been observed, resulting in a global pandemic.^[1,2]^ According to the latest World Health Organization report, approximately 700 million individuals have been infected with COVID-19 worldwide, and nearly 6 million deaths have been recorded.^[3]^ COVID-19 is widely considered one of the most widespread and significant pandemics of the 21st century. COVID-19 is caused by the severe acute respiratory syndrome coronavirus 2 (SARS-CoV-2),^[4,5]^ which binds to angiotensin-converting enzyme 2 receptors on host cells.^[6,7]^ This interaction facilitates virus replication and triggers an inflammatory response that can lead to severe pulmonary inflammation and immune dysregulation.^[8–10]^ Although the clinical manifestations of COVID-19 vary considerably, with most patients being asymptomatic or experiencing only mild symptoms, older adults have higher rates of severe outcomes, including pulmonary inflammation, acute respiratory distress syndrome , and deaths.^[11–14]^ Oncology patients, owing to immunosuppression from both their malignancy and antineoplastic therapies, are particularly vulnerable to severe COVID-19 and tend to experience more severe disease.^[15–21]^

Immune checkpoints are molecules expressed on the cell membranes of immune cells-including programmed cell death receptor-1 (PD-1), programmed cell death ligand-1, cytotoxic T lymphocyte associated antigen-4 (CTLA-4), and T-cell immunoglobulin and mucin-domain containing-3-which are primarily responsible for regulating immune suppression as well as modulating the magnitude and duration of immune responses.^[22,23]^ The function of CTLA-4 is manifested during the initial phase of T-cell activation through the inhibition of self-reactive lymphocytes, thereby preventing autoimmunity.In contrast, PD-1 is instrumental in modulating immune responses within peripheral tissues.^[24–26]^ Under physiological conditions, immune tolerance is defined as the capacity of the immune system to prevent attacks on self-antigens while maintaining controlled responsiveness. However, in cancer, it is plausible that these tolerance mechanisms may be exploited by tumor cells to evade immune surveillance.^[22,27,28]^ Immune checkpoint inhibitors (ICIs) are engineered to abrogate the interactions between checkpoint receptors on immune cells and their ligands, thereby overcoming established immune tolerance.^[29–31]^ Consequently, immune cell-mediated cytotoxicity against cancer cells is restored, and this effect has been associated with significant antitumor outcomes in a range of malignancies, including non-small cell lung cancer,^[32,33]^ melanoma,^[34,35]^ renal cell carcinoma,^[36]^ breast cancer,^[37]^ hepatocellular carcinoma,^[38]^ and head and neck cancer.^[39]^

During the COVID-19 pandemic, the continuation of ICI therapy for cancer patients was advocated by certain scholars. Related mechanistic immunological studies have elucidated a critical commonality between cancer and chronic viral infections (e.g., hepatitis B virus, SARS-CoV-2), demonstrating their pronounced tendency to induce T-cell decline and depletion.^[40–43]^ Consequently, it has been hypothesized by researchers that the immunomodulatory effects of ICI therapy may facilitate viral clearance. Conversely, it has also been postulated by concurrent investigations that ICI therapy may enhance patient susceptibility to infectious processes.^[14,44]^ Relevant clinical evidence substantiates that ICI therapy have the potential to induce immune system overactivation, thereby precipitating immune-related adverse events (irAEs), such as immune pneumonia and immune hepatitis. These complications may significantly worsen the clinical prognosis of cancer patients with concurrent COVID-19 infection.^[45–48]^ This situation underscores the necessity for a cautious and balanced approach when considering ICI therapy in the context of COVID-19.

Despite significant advances in managing the global pandemic, SARS-CoV-2 continues to result in the emergence of novel variants. Currently, a standardized and effective treatment regimen for COVID-19 has not been established.^[49,50]^ Therefore, the retrospective analysis of treatment risks for oncology patients with COVID-19 is imperative. This review summarizes current findings on ICI therapy and COVID-19, thereby offering insights into the clinical characteristics and prognosis of cancer patients receiving ICI therapy concomitant with COVID-19 infection.

## 2. Method

### 2.1. Search strategy

A comprehensive search of the PubMed database was conducted utilizing the following keywords in titles and abstracts: (“immune checkpoint inhibitors” OR “PD-1/PD-L1” OR “CTLA-4” OR “immunotherapy”) AND (“cancer” OR “tumor” OR “neoplasm” OR “malignancy”) AND (“severe acute respiratory syndrome coronavirus 2” OR “SARS-CoV-2” OR “2019-nCoV” OR “COVID-19” OR “2019 Novel Coronavirus”). As of September 2023, 617 English-language articles were identified. After screening abstracts, publications deemed irrelevant (along with conference abstracts, reviews, and meta-analyses) were excluded, leaving 77 relevant articles. A subsequent full-text review resulted in the elimination of duplicate publications and studies with small sample sizes (i.e., those with fewer than 10 participants), resulting in a final selection of 18 articles^[51–68]^ (Table 1).

**Table 1:** Summary of cases of ICI treatment in cancer patients infected with COVID-19.

No	Author	Recruitment period	Country	Age (mean/median)	Sex (male)	Total number	Number of ICIs	Cancer type	Research type	Outcome
1	Rogiers, Aljosja et al	March 5, 2020, to May 15, 2020	Internet	63 (range 27–86)	65%	110	110	Melanoma, NSCLC, RCC	Retrospective cohorts	Hospitalization, mortality
2	Robilotti, Elizabeth V et al	March 10, 2020, to April 7, 2020	USA	≥60 (56%)	50%	423	31	Breast, colorectal, lung, lymphoma	Retrospective cohorts	Hospitalization, severe disease
3	Wu, Qiuji et al	January 9, 2020, to March 20, 2020	China	66 (range 29–73)	73%	11	11	Lung, cervical, endometrial, liver, colorectal	Retrospective cohorts	Severe disease
4	Lee, Lennard Yw et al	March 18, 2020, to April 26, 2020	UK	69 (range 59–76)	56%	800	44	Digestive, hematological, breast	Prospective cohorts	Mortality
5	Guo, Mengni et al	August 2020 to August 2021	USA	67 (range 41–91)	38%	56	56	Thoracic, genitourinary, gynecological	Retrospective cohorts	Hospitalization, severe disease
6	Tan, Ruoding et al	February 20, 2020, to January 28, 2021	USA	66 (SD 13.7)	57%	228	228	Solid, lung, hematological	Retrospective cohorts	Hospitalization, mortality
7	Hatic, Haris et al	January 2020 to November 2021	USA	64 (SD 11.6)	58%	121	61	Lung, liver, renal, HNC	Retrospective cohorts	Hospitalization, mortality
8	Isgrò, Maria Antonietta et al	March 30, 2020, to May 15, 2020	Italy	62 (IQR 53–73)	55%	287	287	Melanoma	Retrospective cohorts	Infection
9	Bui, Ai-Tram N et al	March 20, 2020, to June 3, 2020	USA	72 (range 45–83)	56%	25	25	Thoracic, melanoma, gastrointestinal	Retrospective cohorts	Mortality, IrAEs
10	Klebanov, Nikolai et al	July 1, 2019, to February 29, 2020	USA	67 (SD 12.0)	58%	1545	1545	Hematologic, solid	Retrospective cohorts	Infection, mortality
11	Mandala, Mario et al	March 5, 2020, to May 18, 2020	Italy	66.5 (SD 12.1)	67%	293	159	Melanoma, NSCLC, RCC	Prospective cohorts	Infection, irAEs
12	Guo, Mengni et al	August 2020 to August 2021	USA	67 (range 24–98)	49%	579	579	NSCLC, HCC, RCC, melanoma	Retrospective cohorts	irAEs
13	Bakouny, Ziad et al	March 17, 2020, to May 12, 2022	CCC19	65 (IQR 57–73)	57%	599	599	Solid, hematological	Retrospective cohorts	Severe disease
14	Várnai, Csilla et al	March 18, 2020, to August 1, 2020	UK	72 (range 62–80)	58%	2515	102	Solid, hematological	Prospective cohorts	Mortality
15	Hijmering-Kappelle, L B M et al	January 1, 2019, to June 1, 2021	Netherlands	66 (SD 8.81)	60%	205	205	NSCLC	Retrospective cohorts	irAEs
16	Welti, Michèle et al	February 2020 to February 2022	Switzerland	66 (range 36–86)	60%	25	12	Melanoma	Retrospective cohorts	Severe disease
17	Veron, Marie et al	March 16, 2020, to June 30, 2021.	France	63 (range 55–70)	51%	134	134	NSCLC	Retrospective cohorts	irAEs
18	Chen, Gaili et al	Up to September 30, 2020	China	58 (range 26–77)	79%	24	24	HNC	Retrospective cohorts	Mortality

CCC19 = The COVID-19 and Cancer Consortium, COVID-19 = coronavirus disease 2019, HCC = hepatocellular carcinoma, HNC = head and neck cancers, ICIs = immune checkpoint inhibitors, irAEs = immune-related adverse events, NSCLC = non-small-cell lung cancer, RCC = renal cell carcinoma, SD = standard dosing.

### 2.2. Clinical features

Eighteen articles were selected, of which 15 were retrospective studies,^[51–53,55–60,62,63,65–68]^ and 3 were prospective studies.^[54,61,64]^ The majority of the studies were conducted between 2019 and 2020, predominantly involved populations from Europe and the Americas^[51,52,54–67]^; only 2 studies concentrated on Asian populations.^[53,68]^ The study subjects predominantly consisted of older adults, and the cancer types encompassed melanoma, non-small cell lung cancer, renal cell carcinoma, head and neck cancer, and liver cancer. The investigations primarily examined the risks associated with ICI therapy in cancer patients with COVID-19, specifically concerning infection risk, hospitalization, disease severity, mortality, and potential adverse reactions.

## 3. Result

### 3.1. ICI therapy influencing COVID-19 incidence in cancer patients

Data indicate an increased susceptibility to SARS-CoV-2 infection among cancer patients relative to the general population.^[15,69,70]^ Several studies have assessed the impact of cancer treatments, particularly ICI therapy, on the risk of COVID-19 infection. For instance, a retrospective analysis by Klebanov et al^[60]^ compared 1545 cancer patients receiving ICI therapy with 20,418 control subjects. The infection rates between the groups did not differ significantly (1.4% vs 1.0%, *P* = .16). Moreover, multivariate analysis suggested that ICI therapy did not constitute a significant risk factor for COVID-19 infection (OR 1.38, 95% CI 0.89–2.13, *P* = .15), consistent with other studies.^[45,71,72]^

In contrast, the relationship between ICI therapy and the risk of hospitalization due to COVID-19 is considerably more complex. A retrospective study by Rogiers et al^[51]^ demonstrated that the administration of combined anti-PD-1 and anti-CTLA-4 therapy was associated with increased hospitalization risk among cancer patients diagnosed with COVID-19 (OR 5.68, 95% CI 1.58–20.36, *P* = .027). It is hypothesized that this elevated risk may be attributable to a higher incidence of irAEs (namely excessive inflammation and cytokine storm syndrome) which might contribute to the exacerbation of disease severity. Corroborating this observation, research from the Memorial Sloan Kettering Cancer Center by Robilotti et al^[52]^ identified ICI therapy as an independent risk factor for COVID-19 hospitalization (OR 2.84, 95% CI 1.24–6.72, *P* = .013). Conversely, Tan et al^[56]^ reported no statistically significant difference in hospitalization (38.6% vs 39.0%, *P* = .912) or emergency admission rates (16.7% vs 14.7%, *P* = .50) between patients administered ICI therapy and controls. Although several studies^[52,73,74]^ have examined this issue, the results have remained inconsistent. Evidence also suggests that in specific cancer types (such as hematologic malignancies and lung cancer), ICI therapy may be associated with elevated health risks during the COVID-19 pandemic,^[75–77]^ thereby indicating that treatment strategies warrant careful individualization in these cases.

### 3.2. ICI therapy modulating COVID-19 severity and mortality in cancer patients

Severe COVID-19 is generally defined by requirements for high-flow oxygen therapy, mechanical ventilation, ICU admission, presence of septic shock, or multiple organ dysfunction. Robilotti et al^[52]^ identified ICI therapy as an independent risk factor for severe COVID-19 (OR 2.74, 95% CI 1.37–5.46, *P* = .004). Stratified analyses among patients with lung cancer and other malignancies indicated that ICI therapy may be associated with exacerbated COVID-19 outcomes. The proposed mechanism underlying this association entails that ICIs may induce cytokine storms, exacerbate pulmonary damage, and precipitate acute respiratory distress syndrome via excessive T-cell activation. A case series involving 11 oncology patients in China treated with ICIs was reported by Wu et al^[53]^, revealing that 63.64% of patients developed severe COVID-19, a proportion substantially exceeding that observed in the general population. Nonetheless, other studies have reported disparate findings. For example, no significant association between COVID-19 severity and ICI therapy (OR 0.88, 95% CI 0.42–1.84) or other non-immunotherapeutic cancer treatments (OR 1.10, 95% CI 0.89–1.36) was found by Bakouny et al^[63]^ in an analysis conducted by the COVID-19 and Cancer Consortium. However, among patients with underlying immunosuppression, immunotherapy was associated with an increased likelihood of adverse COVID-19 outcomes (OR 3.33, 95% CI 1.38–8.01). Similarly, no significant correlation between ICI therapy and COVID-19 severity in melanoma patients was reported by Welti et al^[66]^ (*P* = .6).

Recent data indicate that the global average COVID-19 case-fatality rate is approximately 1%^[3]^, although this rate is substantially higher among oncology patients.^[15]^ A prospective study by Lee et al^[54]^ demonstrated that ICI therapy was not associated with a significant elevation in the risk of COVID-19-associated mortality (OR 0.59, 95% CI 0.27–1.27, *P* = .177). Similarly, no significant association between ICI therapy and increased COVID-19 mortality (OR 0.36, 95% CI 0.07–1.87, *P* = .23) was reported by Bui et al^[59]^. Furthermore, a comparison of cancer patients receiving ICIs with those not receiving such therapy, as reported by Tan et al^[56]^, revealed no statistically significant difference in COVID-19-related mortality (overall mortality: 22.4% vs 22.4%, *P* = 1.000; 30-day mortality: 12.7% vs 14.9%, *P* = .235). An elevated mortality rate in patients receiving ICI therapy relative to those not receiving such therapy (40.9% vs 28.6%) was noted by Klebanov et al^[60]^; however, this difference did not reach statistical significance (*P* = .23). Furthermore, multivariate analysis confirmed that ICI therapy was not independently associated with an increased risk of COVID-19-related mortality (OR 1.60, 95% CI 0.78–3.29, *P* = .71).

In summary, available evidence indicates that continuing ICI therapy during the COVID-19 pandemic is not associated with a significant increase in COVID-19-related mortality risk.^[78–80]^ Although ICI therapy may elevate the risk of severe COVID-19 and associated hospitalization via cytokine-mediated immune responses, it may concurrently confer potential therapeutic benefits in severe cases via enhanced immune activation, thereby potentially reducing overall mortality.

### 3.3. ICI therapy comparing prognostic outcomes with alternative cancer treatments

At present, the primary cancer treatment modalities comprise surgery, chemotherapy, radiation therapy, immunotherapy, and targeted therapy.^[81–85]^ During the COVID-19 pandemic, prognoses of cancer patients with COVID-19 may vary by treatment modality. Mandala et al^[62]^ reported that although the COVID-19 infection rate among patients receiving ICI therapy were numerically higher than that in patients receiving chemotherapy and targeted therapy, the difference did not reach statistical significance (32.7% vs 26.0% vs 28.6%; *P* = .610). Similarly, Isgrò et al^[58]^ observed no statistically significant difference in COVID-19 positivity rates between patients receiving ICIs and the general population (3.1% vs 4.6%, *P* = .29); however, ICI therapy was identified as a protective factor against infection after adjustment for gender and cancer stage (OR 0.41, 95% CI 0.18–0.91, *P* = .03). Regarding COVID-19 severity and outcomes, Hatic et al^[57]^ determined that no statistically significant difference in hospitalization rates was observed between the ICIs and chemotherapy groups, although the requirements for mechanical ventilation were higher in patients treated with chemotherapy (3% vs 0%). Although the overall mortality rate was elevated in the ICIs group relative to the chemotherapy group (24.6% vs 13.3%), COVID-19-related fatalities were proportionately lower among ICIs-treated patients (33% vs 50%), and no significant difference in overall survival was observed (780 days vs 706 days; *P* = .6). Mandala et al^[61]^ and Rogiers et al^[51]^ similarly reported no statistically significant difference in COVID-19-related mortality when comparing ICI therapy with chemotherapy, as well as when contrasting ICIs monotherapy with combination therapy. Tan et al^[56]^ corroborated these findings by demonstrating that no statistically significant differences in overall survival or mortality rates between ICIs monotherapy and ICIs in combination with chemotherapy. Additionally, Várnai et al^[64]^ reported that patients administered ICI therapy exhibited significantly lower COVID-19-related mortality than patients receiving no anticancer treatment in the 4 weeks before infection (OR 0.52, 95% CI 0.31–0.86), thereby suggesting a potential protective effect. Moreover, Isgrò et al^[58]^ reported a reduced incidence of immunoglobulin abnormalities in the ICIs group than chemotherapy-treated patients (3.1% vs 6.5%, *P* = .04). In summary, the findings suggest that although ICIs therapy may not substantially increase COVID-19 infection risk or mortality relative to other treatment modalities, it may provide protective benefits concerning both infection risk and COVID-19-related mortality in specific contexts.

### 3.4. ICI therapy duration impacting prognosis in COVID-19 cancer patients

The variation in COVID-19 outcomes attributable to ICI treatment cycles and dosing intervals depends on the specific treatment regimens and patient conditions. A higher proportion of severe COVID-19 was observed among patients receiving ≥3 ICI cycles compared to those receiving fewer cycles, as documented by Wu et al^[53]^ (85.7% vs 25%). However, the limited sample size may have limited the ability to achieve statistical significance (*P* = .09). In contrast, no significant impact on hospitalization rates, severity, or mortality from COVID-19 was reported by Guo et al^[55]^ regarding continuous ICIs exposure, the timing of the last ICIs dose relative to COVID-19 diagnosis, or the total number of treatment cycles. Comparative studies of extended interval (EI) dosing versus standard regimens during the pandemic produced consistent findings. Hijmering-Kappelle et al^[65]^ documented a decreased (albeit statistically nonsignificant) incidence of grade ≥3 adverse events with EI dosing relative to standard dosing (8.9% vs 17.0%, *P* = .42), while Veron et al^[67]^ reported reduced rates of grade ≥3 adverse events and fewer treatment interruptions attributable to adverse reactions among patients receiving EI dosing. It was further elucidated by Chen et al^[68]^ that extended dosing intervals did not appear to exert adverse effects on outcomes in head and neck cancer patients who had achieved complete or partial responses or who had received prolonged prior ICI treatment; however, patients with stable disease may be susceptible to disease progression with extended intervals. In summary, modifying the number of ICI treatment cycles or prolonging dosing intervals does not appear to detrimentally affect COVID-19 prognosis or cancer outcomes, although uninterrupted treatment is recommended for patients manifesting disease progression.

### 3.5. ICI therapy inducing immune-related adverse events in COVID-19 patients

Investigations of the association between COVID-19 infection and irAEs in patients receiving ICI therapy have yielded variable outcomes. Bui et al^[59]^ reported that COVID-19 infection was not associated with a significant increase in the risk of severe irAEs among patients receiving ICIs. Similarly, Mandala et al^[61]^ observed no significant difference in the incidence of low-grade adverse events (25% vs 34.2%, *P* = .9112) or severe adverse events (18.8% vs 3.6%, *P* = .891) between COVID-19-positive and COVID-19-negative patients. Guo et al^[62]^ found no significant difference in overall irAE incidence between COVID-19-positive and COVID-19–negative patients (30.4% vs 19.9%, *P* = .18); however, severe irAEs (grade ≥3) were observed significantly more frequently in patients with COVID-19 infection (10.9% vs 3.2%, *P* = .02). Multivariate analysis determined that COVID-19 infection constituted an independent risk factor for severe irAEs (OR 1.08, 95% CI 1.02–1.14, *P* = .01), while combined ICI therapy (ipilimumab plus nivolumab) was independently associated with an increased risk of irAEs of any grade (OR 1.67, 95% CI 1.39–2.01, *P* < .0001). Furthermore, Veron et al^[67]^ demonstrated that extended-interval ICIs dosing during the pandemic was not associated with a significant increase in the risk of severe irAEs (OR 1.13, 95% CI 0.37–3.79, *P* = .8), and that second-line or subsequent ICIs regimens were not associated with an elevated risk of severe irAEs compared to first-line regimens (OR 0.59, 95% CI 0.18–1.70, *P* = .3). Collectively, these findings suggest that although COVID-19 infection may not substantially influence the incidence of low-grade irAEs, it appears to be associated with an increased risk of severe irAEs. Conversely, the selection of alternative ICIs regimens or the adoption of moderately extended treatment intervals does not appear to significantly affect the incidence of irAEs, although combination ICI therapy is associated with a higher risk.

## 4. Discussion

Since its emergence in late 2019, the COVID-19 pandemic has substantially affected healthcare systems worldwide, particularly impacting cancer patients, who exhibit heightened susceptibility to SARS-CoV-2 infection and related complications. This increased vulnerability is attributable to both malignancy-induced immunosuppression, which may accelerate COVID-19 progression, and to disruptions in cancer care induced by the pandemic, which have collectively worsened patient outcomes.^[15,44,86]^

While ICI therapy has been hypothesized to enhance immune responses against SARS-CoV-2, potentially expediting viral clearance and increasing patient resilience to infection.^[58]^ These putative protective effects must be judiciously weighed against inherent risks. Specifically, ICIs may elicit irAEs, which can exacerbate pulmonary injury and augment susceptibility to severe COVID-19 outcomes. Furthermore, the overlapping clinical presentations of COVID-19 pneumonia and ICI-associated pneumonitis may confound diagnostic processes and impede clinical management.^[87,88]^

In this systematic review, data from 18 global studies investigating the clinical outcomes and prognoses of cancer patients receiving ICIs during the COVID-19 pandemic were analyzed. The findings indicate that ICI therapy is not associated with a significant increase in COVID-19 susceptibility among cancer patients. Nevertheless, among those who develop COVID-19, ICI treatment may be associated with an elevated risk of hospitalization and severe symptoms, particularly in individuals receiving combination ICI therapy or those with preexisting immunosuppression. Importantly, despite these risks, patients treated with ICIs do not demonstrate inferior prognoses and may even experience lower COVID-19-related mortality.

These observations are consistent with previous studies, which have reported an absence of conclusive evidence regarding the association between ICI therapy and increased COVID-19 incidence, hospitalization rates, severity, or mortality. Specifically, Minkove et al^[89]^ reported that prior ICI treatment did not substantially affect COVID-19 prognosis, whereas Liu et al^[79]^ and Ruiz et al^[74]^ observed no significant differences in clinical outcomes between ICI-treated and non-ICI-treated cancer patients. Pan et al^[90]^ further indicated that, although the majority of ICI-treated cancer patients did not exhibit worsened COVID-19 outcomes, combination ICIs regimens may be associated with an elevated risk of severe complications and mortality.

The analysis did not reveal any significant prognostic differences between patients receiving ICI therapy and those receiving chemotherapy while infected with COVID-19. Furthermore, neither the number of ICI treatment cycles nor the interval between the last ICIs administration and the diagnosis of COVID-19 was significantly associated with prognosis. Additionally, temporary suspension or delay of ICI therapy during active COVID-19 infection was not found to adversely affect patient outcomes. Although ICIs were generally not associated with an increased incidence of low-grade adverse events during the pandemic, they may be linked to a higher risk of serious adverse events.

Despite these insights, the review has limitations, primarily pertaining to the underrepresentation of Asian populations in the existing literature, as the majority of the data originate from Europe and the United States. Consequently, the extent to which these findings may be generalized to Asian populations remains uncertain. Future clinical studies, particularly those encompassing Asian patients, are necessary to comprehensively evaluate the efficacy and safety profile of ICI therapy in the context of COVID-19 among diverse demographic groups.

## Author contributions

**Conceptualization:** Kejian Yang.

**Data curation:** Kejian Yang, Zuyin Wan, Junqi Liu, Jusen Nong.

**Investigation:** Haixiang Xie, Xin Zhou, Jianzhu Luo, Chongjiu Qin.

**Methodology:** Kejian Yang, Haixiang Xie.

**Project administration:** Tao Peng.

**Supervision:** Tao Peng.

**Writing – original draft:** Kejian Yang.

**Writing – review & editing:** Kejian Yang, Tao Peng.
